# The Correlation of Optical Coherence Tomography Angiography Parameters With Serum Vascular Endothelial Growth Factor-A (VEGF-A) Levels in Diabetic Macular Edema

**DOI:** 10.7759/cureus.88026

**Published:** 2025-07-15

**Authors:** Punita K Sodhi, Aman S Rana, Divya D, Apoorva Goyal, Miyani Hitenkumar Parvinbhai, Bhawna Mahajan, Bratati Banerjee, Hage Amung, Richa Asthana

**Affiliations:** 1 Ophthalmology, Guru Nanak Eye Centre and Maulana Azad Medical College, New Delhi, IND; 2 Biochemistry, Govind Ballabh Pant Hospital, New Delhi, IND; 3 Community Medicine, Maulana Azad Medical College, New Delhi, IND

**Keywords:** diabetes, diabetic macular edema, optical coherence tomography, optical coherence tomography angiography, vascular endothelial growth factor a levels

## Abstract

Background and objective

Vascular endothelial growth factor-A (VEGF-A) plays an important role in the development of diabetic macular edema (DME). In this study, we aimed to correlate optical coherence tomography angiography (OCTA) parameters with serum VEGF-A levels in DME.

Methodology

We conducted a prospective study, which examined 44 eyes of 32 DME patients (mean age: 56.0 ±9.1 years) for visual acuity (VA), defective colour vision (CV), and contrast sensitivity (CS). The OCT was done for central foveal thickness (CFT), sub-foveal choroidal thickness (SFCT), neurosensory detachment (NSD), epiretinal membrane (ERM), and disorganized retinal inner layers (DRIL). The OCTA was used to study the foveal avascular zone (FAZ) in superficial capillary plexus (SCP) and vessel density (VD) in six retino-choroidal layers, including SCP, deep capillary plexus (DCP), outer retina (OR), outer retinal chorio-capillaries (ORCC), chorio-capillaries (CC), and choroid. The serum VEGF-A levels were measured using an enzyme-linked immunosorbent assay (ELISA) kit. The eyes were treated with a single intravitreal injection of 0.3 mg/0.05 ml ranibizumab biosimilar (RbB). The subjects were re-examined after a short period of one month for visual, OCT, and OCTA parameters and serum VEGF-A levels. A correlation between OCTA parameters and serum VEGF-A was done on both occasions.

Results

At baseline, the logarithm of the minimum angle of resolution (logMAR) best-corrected visual acuity (BCVA) was 0.82 ±0.13, CFT 464.60 ±173.06 microns, SFCT 238.02 ±42.88 microns, and the serum VEGF-A level was 487.94 [interquartile range (IQR): 305 to 775.52] pg/ml. There was a positive correlation of serum VEGF-A level with LogMAR BCVA (r=0.15), CFT (Rho=0.02) and SFCT (Rho=0.14), while there was a negative correlation with FAZ area (Rho=-0.20), perimeter (Rho=-0.19) and VD of SCP (Rho=-0.22), DCP (Rho=-0.02), OR (Rho=-0.10), ORCC (Rho=-0.12), CC (Rho=-0.10) and choroid (Rho=-0.17). After a short follow-up of one month following intravitreal injection, LogMAR BCVA was 0.53 ±0.22, CFT 350.07 ±153.02 microns, SFCT 232.40 ±44.30 microns, and the serum VEGF-A level measured 404.02 (IQR 204.61 to 750.87) pg/ml. There was a positive correlation of serum VEGF-A level with LogMAR BCVA (r=0.48), CFT (Rho=0.19), and SFCT (Rho=0.11), while there was a negative correlation of serum VEGF-A levels with FAZ area (Rho=-0.01), perimeter (Rho=-0.06), and VD of SCP (Rho=-0.22), DCP (Rho=-0.13), OR (Rho=-0.10), ORCC (Rho=-0.25), CC (Rho=-0.18), and choroid (Rho=-0.29). The correlation coefficient values indicated that all observed correlations were weak.

Conclusion

In our study, the BCVA decreased while CFT and SFCT increased, along with an increase in serum VEGF-A levels. The higher serum VEGF-A was related to smaller FAZ and lower retino-choroidal VD. The observed weak correlation of ocular, OCT, and OCTA features with serum VEGF-A levels may limit the predictive value and clinical utility of these biomarkers in the management of DME.

## Introduction

Vascular endothelial growth factor-A (VEGF-A) is an archetypal member of the VEGF family [1]. VEGF-A leads to arteriolar dilatation, increased blood flow, disrupted endothelial barrier, microaneurysm formation, increased vascular permeability, and neovascularization [2]. Patients with diabetic retinopathy (DR) have increased levels of VEGF-A in serum and vitreous [3,4]. These have been shown to correlate with an increased breakdown of the blood-retinal barrier and severity of diabetic macular edema (DME) [1]. Wu et al. stated that aqueous and plasma are more accessible than vitreous, and hence, these can be utilized as surrogate samples for assaying the vitreous [4]. It appears that serum VEGF-A levels can be used to predict the severity of disease and prognosticate outcomes of anti-VEGF therapy. Though VEGF-A is very frequently implicated in angiogenesis in retinal vascular diseases and, therefore, is a therapeutic target, very few studies have measured serum VEGF-A levels in subjects with DME [3-5].

Optical coherence tomography angiography (OCTA) studies the foveal avascular zone (FAZ) area, perimeter, and circularity and measures vascular density (VD) of at least six retino-choroidal layers. It reveals the vessels themselves. Because it is noninvasive, it is widely used to study enlargement and distortion of FAZ, capillary non-perfusion (CNP), and neovascularization in diabetic patients [6]. There is a paucity of literature on the correlation of OCTA parameters in DME with serum VEGF-A level. This study aimed to investigate the correlation between OCTA parameters and serum VEGF-A levels in patients with DME. DR is a retinal vascular disease, and OCTA is relevant in DR as it studies the vasculature of the retina and choroid. The serum VEGF-A levels are raised in DR. Thus, a study on the relation between OCTA parameters and serum VEGF-A levels may aid in understanding the systemic contribution to retinal vascular pathology, potentially improving prognostic assessments and informing therapeutic interventions in DME management.

This study was initially presented as a Free Paper at the Delhi Ophthalmological Society Mid-term Conference 2024, New Delhi, India, on November 23, 2024.

## Materials and methods

We received institutional ethical committee approval vide letter number F.1/IEC/MAMC/MD/MS (92/04/2022/No. 287 dated 29/08/2022, and informed consent was obtained from study participants. The study adhered to the tenets of the Declaration of Helsinki. Treatment-naive eyes with DME were recruited in a prospective interventional study from December 2022 to December 2023. Only those patients with visual acuity (VA) equal to or more than the logarithm of the minimum angle of resolution (logMAR) 1 (6/60, 20/200) were recruited for the study. Subjects with co-existent significant ocular diseases, including glaucoma, media opacity, optic nerve disease, and other retinal diseases, or with a risk of thromboembolic events, were excluded.

Patients were asked about visual complaints such as diminution of vision, defective colour vision (CV), and contrast sensitivity (CS). They were examined monocularly for VA on self-illuminated Early Treatment Diabetic Retinopathy Study (ETDRS) charts at 4 meters under standardized lighting conditions. Further refractive error (RE) and best-corrected visual acuity (BCVA) were recorded. The intraocular pressure (IOP) was measured using Goldman applanation tonometry (AT 020, Carl Zeiss Meditec AG, Jena, Germany). The CV was tested using Ishihara plates (38th edition, 2012) at a distance of approximately 70 cm as well as the Farnsworth test at a distance of approximately 50 cm. The CS was examined on a uniformly illuminated Pelli-Robson chart (Haag-Streit Service, Inc., Mason, Ohio) at 1-meter distance. Each affected eye was tested separately for CV and CS, under ambient light conditions, with subjects wearing appropriate refractive correction.

Following dilatation, we examined the fundus, captured a color fundus photograph, and took subjects for fundus fluorescein angiography on a fundus camera (FF450plus fundus camera Visucam PRO NM, Carl Zeiss Meditec AG). A 3 x 3 mm optical coherence tomography (OCT) scan (SD‐OCT RTVue, Optovue Inc., Fremont, CA) of central 30 degrees around the fovea examined central foveal thickness (CFT), neurosensory detachment (NSD), sub-foveal choroidal thickness (SFCT), and other OCT features of DR. We diagnosed DME in subjects who had CFT measuring greater than 300 microns.

We performed a 3 x 3 mm macular OCTA scan (SD‐OCT RTVue, Optovue Inc., Fremont, CA) to examine FAZ and six retino-choroidal layers. At a rate of 85,000 A-scans per second, the device produced two OCT volumes consisting of 304 x 304 A-scans each in around three seconds. Prototype OCTA software applied a split-spectrum amplitude-decorrelation angiography (SS-ADA) algorithm from these volumes to produce OCT angiograms. The OCTA software automatically segmented the retina and choroid layers, examining the size and regularity of the FAZ. We measured the FAZ area, perimeter, and circularity. The fovea was defined as the area within the central 0.5 mm ring of the ETDRS grid. The parafovea was considered as the area between the central 0.5 mm and the 1.5 mm ring of the ETDRS grid, and the perifovea as the area between the central 1.5 mm and the 3 mm ring of the ETDRS grid.

The VD of six retino-choroidal layers, including the superficial capillary plexus (SCP), deep capillary plexus (DCP), outer retina (OR), outer retinal chorio-capillaries (ORCC), chorio-capillaries (CC), and choroid (C), was automatically generated by the instrument. The SCP was bracketed between the internal limiting membrane (ILM) and the inner plexiform layer/inner nuclear layer (IPL/INL), i.e., between ILM and 13 microns below IPL/INL. The DCP was segmented between IPL/INL and outer plexiform layer/outer nuclear layer (OPL/ONL), i.e., at a depth starting at 8 microns below IPL/INL till 13 microns below OPL/ONL. The OR spanned from the OPL/ONL to the retinal pigment epithelium/Bruch’s membrane (RPE/BM), covering a depth starting at 8 microns below OPL/ONL till 71 microns above RPE/BM. The ORCC extended between OPL/ONL and RPE/BM (i.e., at depth, starting at 8 microns below OPL/ONL and extending to 21 microns below RPE/BM). The CC layer extended from 4 microns to 32 microns beneath the RPE/BM. The choroid was segmented from 25 microns to 63 microns below the RPE/BM. We calculated the VD as the proportion of the measured area occupied by blood vessels with detectable flow, defined as pixels having decorrelation values above a specified threshold. The segmentation errors in automatically acquired scans were manually corrected to enhance image quality, wherever required.

A standardized imaging protocol was used to obtain the OCT and OCTA scans, which were performed on the affected eye by a trained operator under consistent mesopic light conditions. The scans having projection or motion artifacts, like vessel doubling or edge duplication, were excluded. All images were taken at a signal strength index of 90% and a signal quality index of 4/5. Two independent readers (PKS and ASR) measured all parameters, and the readings obtained by them were averaged.

The 3-millilitre blood samples were drawn by venous puncture with minimal stasis for all the subjects, and blood was collected in tubes containing EDTA. After allowing the blood to clot at room temperature for 30 minutes, the sample was centrifuged at 3000 rpm for 15 minutes to remove the clot. The serum was separated and stored at -80 °C. Following a single freeze-thaw cycle, all serum samples were analyzed simultaneously using a commercially available kit (Human VEGF-A ELISA kit, ELK Technology, Cat: ELK 1129, Denver, CO) with an enzyme-linked immunosorbent assay (ELISA) test performed by the same clinician (BM).

Before intervention, the laboratory tests for fasting and postprandial blood sugar, glycosylated haemoglobin (HbA1c), liver function tests, and kidney function were confirmed to be normal. For treating DME, the patients received intravitreal injections of 0.3 mg/0.05 ml ranibizumab biosimilar (RbB; Razumab; Intas, Ahmedabad, India) in sterile conditions inside the operating theatre. The injection was given about 3.5 to 4.0 mm posterior to the limbus with a 30-gauge needle. After removing the needle, a sterile cotton-tip applicator was used to prevent reflux. Postoperatively, tobramycin eye drops were given four times a day. Following anti-VEGF injection, a change in IOP was measured at one week, and a change in visual, OCT, and OCTA parameters was measured at one month. None of the subjects exhibited anterior segment reaction, development or progression of cataract, vitritis, endophthalmitis, vasculitis, retinal haemorrhages, necrosis or retinal detachment.

A repeat analysis for serum VEGF-A was done at one month post-treatment. We assessed the correlation between ocular, OCT, and OCTA parameters and serum VEGF-A levels both before and after RbB injection.

Sample size calculation

According to the available literature (Elnahry et al.) [7], the mean VD before anti-VEGF was 31.3 ±5.58, and the mean VD after anti-VEGF was 28.8 ±5.18. To have a confidence level of 95% and a power of study of 80%, we estimated a sample size of 37 using the following formula:

N = [(Zα+Zβ) σ]^2^/δ^2^

However, we ultimately included 44 eyes in this study.

Statistical analysis

Statistical analysis was performed using SPSS Version 25.0 Software (IBM Corp., Armonk, NY). The primary objective was to measure OCTA parameters before and after intravitreal RbB injection. The secondary objectives were to measure ocular parameters, OCT parameters, and serum VEGF-A levels, before and after treatment, and to correlate ocular, OCT, and OCTA parameters with serum VEGF-A levels. The continuous variables were presented as mean ±standard deviation (SD). Only five features were parametric, including VA, BCVA, IOP, the number of Ishihara plates 1-21 read, and the number of Ishihara plates 22-25 read. The rest of the data was non-parametric. Hence, correlation was found using Pearson (r) and Spearman (Rho) correlation coefficients for parametric and non-parametric data, respectively. The correlation was considered significant only when the p-value was less than 0.05.

## Results

The study included 44 eyes of 32 patients, including 16 males (50%) and 16 females (50%), with a mean age of 56.0 ±9 years. Among the 44 eyes, the disease affected the right eye in 23 (52.27%) and the left eye in 21 (47.42%) patients. The disease duration was defined as the period from the first diagnosis of diabetes mellitus to the time of serum sample collection. The average duration of the disease was 240.22 ±97.74 (60-365) days. Among 44 eyes, 22 (50%) had moderate non-proliferative diabetic retinopathy (NPDR) and 22 (50%) had severe NPDR.

The mean fasting blood sugar was 141.59 ±56.03 mg/dl, postprandial blood sugar was 191.84 ±73.39 mg/dl, HbA1C was 7.53 ±1.33%, blood urea 30.14 ±9.20 mg/dl, serum creatinine was 0.91 ±0.23 mg/dl, serum glutamic-oxaloacetic transaminase was 27 ±13.81 units per litre, and serum glutamic pyruvic transaminase was 22.75 ±6.62 units per litre. The subjects presented with diminution of vision, metamorphopsia, and scotoma. At a pre-treatment stage, the mean VA was LogMAR 0.89 ±0.13, the mean RE was minus 0.5 ±0.9, and the mean BCVA was LogMAR 0.82 ±0.13. The mean IOP was 16 ±1.94 mmHg, and the mean CS was 1.0 ±0.5. The mean number of Ishihara plates 1-21 read at pre-treatment was 18.8 ±1.64, while the mean number of plates 22-25 read at pre-treatment was 3.7 ±0.64. The Farnsworth test showed normal CV in 14 eyes, mild tritanopia in 10, moderate tritanopia in five, severe tritanopia in 12, and deuteranopia in three eyes.

The mean CFT was 464.60 ±173.06 microns, and the mean SFCT was 238.02 ±42.88 microns. All the eyes had DME located within 300 microns of the fovea. Of the 44 eyes, 19 had NSD. Some eyes had even two or three NSDs. Thus, the mean number of NSDs was 0.90 ±0.40 (0 to 3). The mean values for height and width of NSD were 55.56 ±93.39 microns and 466.91 ±691.91 microns, respectively. A total of 44 eyes had edema within 300 microns of the fovea, 43 eyes had intra-retinal cysts, 42 eyes had hard exudates (HE), five eyes had epiretinal membrane (ERM), four eyes had disorganized retinal inner layers (DRIL), and nine eyes had posterior hyaloid thickening (PHT).

Table 1 shows the correlation of serum VEGF-A levels with ocular and OCT features at the pre-treatment stage.

**Table 1:** Correlation of serum VEGF-A levels with ocular and optical coherence tomography features at the pre-treatment stage ^*^Parametric data. ^**^Statistically significant p<0.05. Pearson correlation coefficient was calculated for parametric data, and Spearman correlation coefficient for non-parametric data BCVA: best-corrected visual acuity; DRIL: disorganized retinal inner layers; ERM: epiretinal membrane; LogMAR: logarithm of the minimum angle of resolution; PHT: posterior hyaloid thickening; VA: visual acuity

S. no.	Parameter	Correlation coefficient value	P-value
1.	VA (LogMAR)^*^	0.28	0.06
2.	Refractive error (spherical equivalent)	0.01	0.91
3.	BCVA (LogMAR)^*^	0.15	0.30
4.	Intraocular pressure^*^	-0.18	0.22
5.	Contrast sensitivity	-0.30	0.04^**^
6.	Number of Ishihara 1-21 plates read^*^	-0.02	0.85
7.	Number of Ishihara 22-25 plates read^*^	-0.15	0.32
8.	Central foveal thickness	0.02	0.88
9.	Sub-foveal choroidal thickness	0.14	0.33
10.	Neurosensory detachment number	0.13	0.36
11.	Neurosensory detachment height	0.25	0.32
12.	Neurosensory detachment width	0.20	0.44
13.	Number of eyes with edema within 300 microns	(All eyes had this finding)	-
14.	Number of eyes with cysts	-0.10	0.51
15.	Number of eyes with hard exudates	0.18	0.22
16.	Number of eyes with ERM	-0.26	0.08
17.	Number of eyes with DRIL	-0.21	0.16
18.	Number of eyes with PHT	-0.24	0.11

Among ocular features, serum VEGF-A correlated negatively with CS, IOP, and the number of Ishihara plates read in both categories, i.e., 1-21 and 22-25. In contrast, it correlated positively with the LogMAR value of VA, BCVA, and RE. The OCT features, including CFT, SFCT, NSD number, height, and width, and the number of eyes with HE, had a positive correlation with serum VEGF-A levels. In contrast, the number of eyes with cysts, ERM, DRIL, and PHT had a negative correlation. Only one correlation value was significant, i.e., negative correlation of serum VEGF-A levels with CS (Rho=-0.30; p=0.04).

Table 2 shows the correlation of serum VEGF-A levels with OCTA features at the pre-treatment stage.

**Table 2:** Correlation of serum VEGF-A levels with optical coherence tomography angiography features using Spearman correlation coefficient at the pre-treatment stage ^**^Statistically significant p<0.05 CC: chorio-capillaries; DCP: deep capillary plexus; FAZ: foveal avascular zone; OR: outer retina; ORCC: outer retinal chorio-capillaries; SCP: superficial capillary plexus; VD: vessel density

S. no.	Parameter	Correlation coefficient value	P-value
1.	FAZ area	-0.20	0.18
2.	FAZ perimeter	-0.19	0.20
3.	FAZ circularity	0.01	0.98
4.	SCP foveal VD	-0.07	0.61
5.	SCP parafoveal VD	-0.22	0.14
6.	SCP perifoveal VD	-0.23	0.13
7.	SCP whole VD	-0.22	0.14
8.	DCP foveal VD	-0.17	0.25
9.	DCP parafoveal VD	-0.06	0.69
10.	DCP perifoveal VD	-0.04	0.77
11.	DCP whole VD	-0.02	0.87
12.	OR foveal VD	-0.02	0.86
13.	OR parafoveal VD	-0.09	0.52
14.	OR perifoveal VD	-0.15	0.31
15.	OR whole VD	-0.10	0.50
16.	ORCC foveal VD	-0.14	0.34
17.	ORCC parafoveal VD	-0.13	0.38
18.	ORCC perifoveal VD	-0.11	0.45
19.	ORCC whole VD	-0.12	0.43
20.	CC foveal VD	-0.08	0.56
21.	CC parafoveal VD	-0.11	0.45
22.	CC perifoveal VD	-0.08	0.56
23.	CC whole VD	-0.10	0.50
24.	Choroid foveal VD	-0.22	0.14
25.	Choroid parafoveal VD	-0.21	0.15
26.	Choroid perifoveal VD	-0.12	0.41
27.	Choroid whole VD	-0.18	0.24

The FAZ area, perimeter, and VD in six retino-choroidal layers had a negative correlation with pre-treatment serum VEGF-A levels, while FAZ circularity had a positive correlation. None of the correlations was statistically significant. The blood sample analysis at baseline showed that the median value of serum VEGF-A levels was 487.94 [interquartile range (IQR) 305-775.52] pg/ml, while the mean value was 539.11 ±336.44 (51.23-1243.5) pg/ml. One month after intravitreal injections of RbB, the OCTA parameters and VEGF-A levels were measured again to find a correlation.

At the post-treatment stage, the mean VA improved to LogMAR 0.59 ±0.33; the mean RE improved to minus 0.33 ±0.73, i.e., eyes became less myopic, BCVA improved to LogMAR 0.53 ±0.22, and the mean CS improved to 1.48 ±0.46. The mean IOP decreased to 12.14 ±2.36 mmHg. At post-treatment, there was an increase in the mean number of Ishihara plates read in both categories, i.e., 1-21 plates and 22-25 plates (19.64 ±1.43 and 3.70 ±0.64, respectively). The simultaneous examination with the Farnsworth test showed normal CV in 25 eyes, mild tritanopia in nine, moderate tritanopia in nine, and deuteranopia in one eye.

Following treatment, the mean CFT decreased to 350.07 ±153.02 microns, and the mean SFCT decreased to 232.40 ±44.30 microns. Among 44 eyes, 14 had one, two, or three NSD; the mean NSD number also decreased to 0.36 ±0.65. The mean values for height and width of NSD decreased to 49.68 ±97.87 microns and 376.5 ±709.75 microns, respectively. Following treatment, the number of eyes having edema within 300 microns was 41, that with cystic spaces was 38, HE was 42, ERM was two, DRIL was two, and PHT was two. The blood sample analysis showed that the median value of serum VEGF-A level was 404.02 (IQR: 204.61 to 750.87) pg/ml, while the mean value was 505.96 ±435.90 (31.25-1823.3) pg/ml. The difference between the median values of pre-treatment plasma VEGF A levels and post-treatment plasma VEGF A levels was statistically not significant (p=0.19).

Table 3 shows the correlation of serum VEGF-A levels with ocular and OCT features at the post-treatment stage.

**Table 3:** Correlation of serum VEGF-A levels with ocular and optical coherence tomography features at post-treatment stage ^*^Parametric data. ^**^Statistically significant (p<0.05); Pearson correlation coefficient was calculated for parametric data and Spearman correlation coefficient for non-parametric data BCVA: best-corrected visual acuity; DRIL: disorganised retinal inner layers; ERM: epiretinal membrane; LogMAR: logarithm of the minimum angle of resolution; PHT: posterior hyaloid thickening; VA: visual acuity

S. No.	Parameter	Correlation coefficient value	p value
1.	Visual Acuity (LogMAR)^*^	0.44	0.002^**^
2.	Refractive error (Spherical Equivalent)	-0.11	0.44
3.	Best corrected visual acuity (LogMAR)^*^	0.48	0.001^**^
4.	Intraocular pressure^*^	-0.24	0.11
5.	Contrast sensitivity	-0.44	0.003^**^
6.	Number of Ishihara 1-21 plates read^*^	-0.44	0.003^**^
7.	Number of Ishihara 22-25 plates read^*^	-0.51	0.000^**^
8.	Central foveal thickness	0.19	0.20
9.	Sub-foveal choroidal thickness	0.11	0.45
10.	Neurosensory detachment number	0.23	0.13
11.	Neurosensory detachment height	0.03	0.91
12.	Neurosensory detachment width	-0.15	0.61
13.	Number of eyes with edema within 300 microns	-0.16	0.28
14.	Number of eyes with cysts	-0.25	0.10
15.	Number of eyes with hard exudates	-0.15	0.30
16.	Number of eyes with ERM	-0.12	0.40
18.	Number of eyes with DRIL	-0.35	0.01^**^
19.	Number of eyes with PHT	0.03	0.82

Post-treatment, there was a positive correlation between the LogMAR values of VA and BCVA and serum VEGF-A levels. There was a negative correlation of serum VEGF-A levels with RE, IOP, CS, and the number of Ishihara plates read in both categories, i.e., 1-21 and 22-25. The CFT, SFCT, NSD number, NSD height, and number of eyes with PHT correlated positively, while NSD width and number of eyes with cysts, HE, ERM, and DRIL correlated negatively with serum VEGF-A levels.

The correlation was significant only for VA (LogMAR) (r=0.44; p=0.002), BCVA (LogMAR) (r=0.48; p=0.001), CS (Rho=-0.44; p=0.003), number of Ishihara plates read in both categories (plates 1-21; r=-0.44; p=0.003 and plates 22-25; r=-0.51; p=0.000) and number of eyes with DRIL (Rho=-0.35; p=0.01).

Table 4 shows the correlation of serum VEGF-A levels with OCTA features at the post-treatment stage.

**Table 4:** Correlation of serum VEGF-A levels with optical coherence tomography angiography features using Spearman correlation coefficient at post-treatment stage ^*^Statistically significant p<0.05 CC: chorio-capillaries; DCP: deep capillary plexus; FAZ: foveal avascular zone; OR: outer retina; ORCC: outer retinal chorio-capillaries; SCP: superficial capillary plexus; VD: vessel density

S. no.	Parameter	Correlation coefficient value	P-value
1.	FAZ area	-0.01	0.91
2.	FAZ perimeter	-0.06	0.69
3.	FAZ circularity	-0.05	0.72
4.	SCP foveal VD	0.11	0.44
5.	SCP parafoveal VD	-0.01	0.92
6.	SCP perifoveal VD	-0.004	0.98
7.	SCP whole VD	-0.02	0.85
8.	DCP foveal VD	0.09	0.52
9.	DCP parafoveal VD	-0.02	0.88
10.	DCP perifoveal VD	-0.19	0.21
11.	DCP whole VD	-0.13	0.39
12.	OR foveal VD	-0.16	0.28
13.	OR parafoveal VD	-0.11	0.47
14.	OR perifoveal VD	-0.07	0.61
15.	OR whole VD	-0.10	0.51
16.	ORCC foveal VD	-0.19	0.19
17.	ORCC parafoveal VD	-0.23	0.11
18.	ORCC perifoveal VD	-0.29	0.04^*^
19.	ORCC whole VD	-0.25	0.09
20.	CC foveal VD	-0.14	0.33
21.	CC parafoveal VD	-0.20	0.18
22.	CC perifoveal VD	-0.17	0.24
23.	CC whole VD	-0.18	0.21
24.	Choroid foveal VD	-0.21	0.16
25.	Choroid parafoveal VD	-0.31	0.03^*^
26.	Choroid perifoveal VD	-0.26	0.08
27.	Choroid whole VD	-0.29	0.05

After treatment, all FAZ features and VD in all layers except SCP foveal and DCP foveal correlated negatively with serum VEGF-A levels. At the same time, it was significant for ORCC perifoveal, choroid parafoveal, and choroid whole. The correlation was significant only for ORCC perifoveal VD (Rho=-0.29; p=0.04) and choroid parafoveal VD (Rho=-0.31; p=0.03). For all the parameters, the correlation coefficient value was very low, indicating uniformly weak correlations.

## Discussion

Among the members of the VEGF family, VEGF-A is currently the most implicated in the pathogenesis of various retinal conditions [6]. Still, the serum levels of this cytokine have been studied infrequently. Wu et al. found plasma VEGF-A levels to be 168 (74.6, 262) pg/ml in diabetic patients and 141 (31.8, 315) pg/ml in controls [4]. Mesquita et al. found serum VEGF-A levels of 58.73 pg/ml in diabetic subjects (including NPDR and PDR) vis-a-vis 97.63 pg/ml in controls. Among diabetic subjects, notably, the levels were higher in NPDR (95.08 ±47.37 pg/ml) as compared to PDR (36.02 ±28.05 pg/ml) (p=0.028). The vitreous levels did not correlate with serum levels of VEGF-A, and in PDR, NPDR, and controls, these were 576.88 ±681.15 pg/ml, 39.11 ±71.13 pg/ml, and 7.04 ±1.35 pg/ml, respectively [3]. Neither of these studies found the relationship between serum VEGF-A levels and OCTA features.

Krizova et al. found that serum VEGF levels (414.3, IQR: 293.1-512.0) pg/ml in subjects having moderate to severe NPDR were higher than controls (332.7, IQR: 149.4-551.8) pg/ml. The serum levels did not correlate with OCT parameters, including CFT, cube average thickness, cube volume, and serous retinal detachment [5]. Our study also showed a weak correlation. While Lee et al. attributed the severity of DR to the circulating systemic VEGF-A, Burgos et al. and Funatsu et al. observed that the development of edema and neovascularization are dependent mainly on the intraocular synthesis of VEGF-A [8-10].

This study aimed to find a correlation between various ocular parameters and serum VEGF-A levels in DME before and after RbB injection. The higher VEGF-A levels were associated with decreased visual functions like VA, BCVA, CS, and CV, though the correlation we found was not significant. VEGF receptor 2 is the primary signalling receptor and is strongly expressed by Müller cells and photoreceptors [11]. Hu et al. found that intravitreal injections of VEGF caused a significant reduction of scotopic ERG a-wave and b-wave amplitudes and photopic ERG b-wave amplitudes, in a dose-dependent manner. Thus, VEGF appears to be a direct functional regulator of photoreceptors, and its up-regulation in DR contributes to diabetes-induced alteration of photoreceptor function [12].

There was a positive correlation between VEGF-A and RE before RbB injection. The VEGF receptors on the ciliary body (CB) influence its vascularity and aqueous humor secretion, and the resulting increased secretion inhibits CB contraction, causing ametropia [13]. VEGF-A is a multifunctional cytokine that causes an increased conventional aqueous outflow facility through action on VEGF receptors [14]. Additionally, it is an inflammatory factor. Thus, IOP shows a reduction with an increase in its levels. The vascular hyperpermeability and leakage into the retina and choroid lead to an increased retinal thickening, increased CFT and NSD, and dilation of the vessels in the choroidal layer, causing a thicker choroid [15,16]. The VEGF also induces an increase in blood flow, which causes vascular hyperpermeability and leakage into the retina and choroid [15,17,18]. However, OCTA-derived parameters in our study revealed a negative correlation between serum VEGF-A levels and VD across different retino-choroidal layers.

In DME, an increase in inflammatory factors like cytokines has an additive adverse effect with the inflammatory effect of VEGF, leading to microangiopathy and a reduction in VD [19]. Some authors have used both serum and vitreous samples to study VEGF-A levels [20], but accessing serum samples is definitely easier and has lower risks. In this study, we analyzed serum samples to assess VEGF-A levels. There was a difference in the correlation pattern at the pre-treatment and post-treatment stages for some OCT features. A rise in VEGF caused a decrease in cysts, ERM, and DRIL both at pre-treatment and post-treatment. The VEGF levels correlated positively with NSD width and several eyes with HE and negatively with several eyes with PHT at pre-treatment. In contrast, the opposite pattern was seen after RbB injection. Additionally, while all eyes had edema within 300 microns of the fovea at pre-treatment, only 41 had this after intravitreal injection.

Both the FAZ area and perimeter had a negative correlation with serum VEGF-A levels, suggesting that increasing VEGF causes an arteriolar dilatation and increased blood flow in SCP and DCP. This is further substantiated by the fact that VD in SCP foveal and DCP foveal was seen to correlate positively with VEGF-A levels at least at the post-treatment stage. Otherwise, VD in other retino-choroidal layers showed a reduction pattern with increased serum VEGF-A. The FAZ circularity correlated positively before injection but negatively after injection.

In our study, a single injection of RbB resulted in a slight reduction in serum VEGF-A levels, from a median value of 487.94 pg/ml to 404.02 pg/ml, which was statistically insignificant (p=0.19). It appears that an intraocular VEGF reduction may not translate to systemic levels. However, the difference in the correlation pattern between VEGF-A and some parameters at two time points of measurement (that is, before and after RbB injection) suggests that RbB may also be changing the chemical nature of VEGF-A to some extent. However, no biochemical analysis of VEGF-A was performed in our study to support this speculation.

Limitations

Our study's limitations include a smaller sample size, a shorter treatment period involving only a single RbB injection, a follow‐up period of only one month, and the absence of a control group in a single-center study. The serum VEGF-A levels in DME showed weak and mostly non-significant correlations with ocular, OCT, and OCTA features. We could not readily explain the unexpected negative correlations between OCT features like cysts, HE, ERM, DRIL, and PHT, and VEGF-A levels. We believe this may be because vascular changes mediated by VEGF-A do not directly influence these structural parameters. Serum VEGF-A levels may not adequately reflect intraocular VEGF status, and hence aqueous or vitreous sampling may be required for a more precise assessment. The interpretation could be confounded by variability in glycemic control, disease duration, systemic factors, and individual differences. Additionally, the OCTA equipment we used obtained only 3 × 3 mm scans. All these factors are significant limitations affecting conclusions.

However, despite these limitations, a key strength of this prospective study was the comprehensive correlation of serum VEGF-A levels with seven ocular, 12 OCT, and 27 OCTA parameters at both pre-treatment and post-treatment stages. A detailed and noninvasive examination of six different retino-choroidal layers was done with OCTA. We recommend further studies with larger sample sizes, involving multiple RbB injections and correlation analyses at multiple time points, utilizing both serum and vitreous samples. Additional research utilizing robust statistical methods to adjust for potential confounders may provide more reliable insights.

## Conclusions

The correlation of ocular, OCT, and OCTA parameters with serum VEGF-A level in subjects of DME has been rarely studied. We found a reduction in VA, BCVA, CS, CV, and IOP with increased serum VEGF-A. The serum VEGF-A caused an increase in CFT, SFCT, NSD number, and height. The negative correlations of OCT features such as cysts, HE, ERM, DRIL, and PHT with VEGF-A levels, observed at one or more time points, remain unexplained. A reduction in FAZ area, FAZ perimeter, and VD in different retino-choroidal layers was also observed. It should be noted that there was a weak correlation between various ocular, OCT, and OCTA features and serum VEGF-A levels in DME; the majority of correlations were not statistically significant. The limited strength of correlations observed may limit the predictive value and clinical utility of these biomarkers in the management of DME.
